# ﻿Diversity of *Rhyacophila* (Trichoptera, Rhyacophilidae) in the Hengduan Mountains

**DOI:** 10.3897/zookeys.1263.153111

**Published:** 2025-12-10

**Authors:** Sonja Gerwin, Xiling Deng, Fengzhi He, Steffen U. Pauls

**Affiliations:** 1 Senckenberg Research Institute and Natural History Museum Frankfurt, Senckenberganlage 25, 60325 Frankfurt am Main, Germany Senckenberg Research Institute and Natural History Museum Frankfurt Frankfurt am Main Germany; 2 Senckenberg Biodiversity and Climate Research Centre, Senckenberganlage 25, 60325 Frankfurt am Main, Germany Senckenberg Biodiversity and Climate Research Centre Frankfurt am Main Germany; 3 LOEWE Centre for Translational Biodiversity Genomics, Senckenberganlage 25, 60325 Frankfurt am Main, Germany LOEWE Centre for Translational Biodiversity Genomics Frankfurt am Main Germany; 4 Institute of Insect Biotechnology, Justus-Liebig-University Gießen, Heinrich-Buff-Ring 26-32, 35392 Gießen, Germany Justus-Liebig-University Gießen Gießen Germany; 5 Leibniz Institute of Freshwater Ecology and Inland Fisheries, Müggelseedamm 310, 12587 Berlin, Germany Leibniz Institute of Freshwater Ecology and Inland Fisheries, Müggelseedamm Berlin Germany; 6 Northeast Institute of Geography and Agroecology, Chinese Academy of Sciences, Shengbei Street 4888, 130102 Changchun, China Northeast Institute of Geography and Agroecology, Chinese Academy of Sciences Changchun China

**Keywords:** Caddisflies, mountain biodiversity, species delimitation

## Abstract

Aquatic insects are particularly dependent on environmental conditions because their life cycles are directly linked to physico-chemical conditions in freshwater habitats. Here, we combine DNA barcoding and ecological analysis to determine general distributional patterns for an unknown fauna of *Rhyacophila* (Trichoptera, Rhyacophilidae) in the Hengduan Mountains in China. In total 415 larval and 109 adult specimens from four major Hengduan Mountain river basins (1022 m – 4381 m a.s.l.) were sequenced and analyzed. Molecular operational taxonomic units (MOTUs) were delimited as putative species analogs. MOTUs were derived from mitochondrial COI (mtCOI) and nuclear wingless (nuWG) data using the tree-based GMYC method. As expected, based on a higher mutation rate, mtCOI delimitation resulted in a much higher number of MOTUs (66) than in WG (27), but not all mtCOIMOTUs were nested within nuWGMOTUs. Many mtCOIMOTUs were geographically restricted and rare, often occurring in only one or two sampling sites. Multivariate GLM analyses confirmed the importance of basins as the primary factor for explaining the diversity of *Rhyacophila*MOTUs. The high level of geographical restriction of MOTUs among basins and along elevational gradients indicates that *Rhyacophila* in the Hengduan Mountains are largely range-restricted, dispersal limited and consequently vulnerable to local changes in environmental conditions.

## ﻿Introduction

Due to high levels of habitat heterogeneity, mountains generally host high levels of biological diversity, even over short geographic distances. These patterns are frequently considered to be governed by high gradient topography that leads to clear elevational distribution patterns and topographic dispersal barriers (Favre et al. 2015). In caddisflies, these dispersal barriers can act at either the larval or adult dispersal phases or both. Larval dispersal is limited inside the stream, primarily consists of downstream movement, and should lead to diversification among watersheds (Headwater Pattern, *sensu*Hughes et al. 2009). However, the upstream movement of flying adults is not restricted to the river valley. While upstream river corridor dispersal is common and important to compensate for downstream larval drift, lateral dispersal between catchments or basins is regularly observed and inferred (e.g., Geismar et al. 2015).

In the extremely deep parallel river valleys of the Hengduan Mountains in south-western China, we might expect lateral movement to be restricted due to the extremely high mountains separating neighboring valleys. This restriction would presumably lead to highly structured caddisfly distributions, valley-specific differentiation and even speciation (Malicky 2004; Hoppeler et al. 2016). This, in turn, would lead to a high number of endemic lineages within river valleys in the region. At the same time, the extensive elevational gradients across the mountain range lead to a plethora of environmental conditions across relatively short geographic distances within the river network, potentially driving ecological diversification and specialization (Favre et al. 2015).

Here we assess the elevational and network-associated diversity patterns of *Rhyacophila* species in streams and rivers in the Hengduan Mountains. We predict that valley system endemics will dominate the overall diversity patterns. Considering the high elevational gradients in these rivers from ~1000 m a.s.l. to >4300 m a.s.l., we further expect to see elevational diversification in response to varying environmental conditions from subtropical to alpine conditions.

## ﻿Materials and methods

### ﻿Study sites

**Figure 1.**
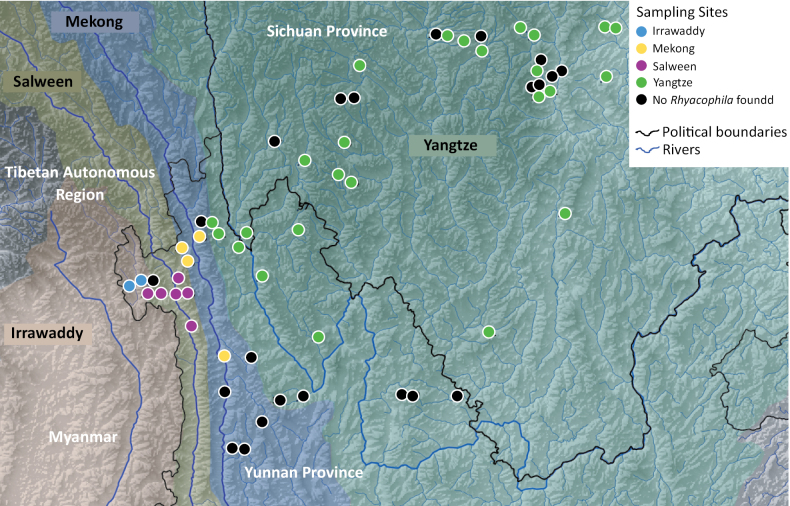
Distribution of sampling sites in the four major drainage basins of the Hengduan Mountains.

Larval and adult specimens were collected in the Yangtze (33 sites), Salween (7 sites), Mekong (6 sites) and Irrawaddy (3 sites) basins (see Fig. 1) by Xiling Deng and Fengzhi He in 2018. Sampling methods were described in detail in Hjalmarsson et al. (2018). In short, larval specimens were collected by hand picking from rocky substrates or collected by kick-sampling and hand nets. Adults were collected using aerial hand nets and light trapping with passive pan traps.

### ﻿Environmental variables

At each site, elevation [m], conductivity [µs], salinity [ppt], total amount of dissolved solids (TDS) [g/L], water temperature [°C], air temperature [°C], dissolved oxygen [mg/L], dissolved oxygen [%], pH and the oxidation reduction potential [mV] were measured using a multiparameter water quality meter (YSI professional plus, US). Altitude and the exact geographical position were determined with a GPS device (Garmin Etrex, Schaffhausen).

### ﻿Identification and preparation of specimens

The inspection of the larvae and sorting into morphospecies was done using an Olympus SZX7 stereoscope with a mounted DP25 5MP camera. To identify the adults to species/genus level, one or two male individuals per morphospecies were selected and the genitals were cleared with lactic acid (Blahnik et al. 2007). Identifications were based on Morse et al. (1994) for larvae and unpublished species pages from H. Malicky (Lunz am See, Austria) and Schmid 1970 for adults. One or two hind legs of each individual were removed for DNA extraction.

### ﻿DNA extraction, amplification, and sequencing

We extracted DNA from all *Rhyacophila* specimens collected. In total 524 specimens of *Rhyacophila* were included in this study (415 larvae, 109 adults; 1–43 specimens per site). Genomic DNA of each specimen was extracted using hindlegs and/or abdominal tissue with the HotShot protocol (Montero-Pau et al. 2008). We amplified and sequenced two gene fragments to complement morphospecies assignment by means of integrative species delimitation: the COI barcode region (658bp) and the nuclear gene wingless (472bp). PCR and sequencing primers are listed in Table 1. PCR amplification of the mtDNA COI was performed with 2.0 µl genomic DNA in 11µl reactions, using the following recipe: 2.0 µl of reaction Buffer (5XMy Taq Red Reaction Buffer, meridian bioscience), 0.4 µl of each primer, 0.1 µl Taq DNA Polymerase (My Taq DNA Polymerase, meridian bioscience) and 6.1 µl of sterile water. PCR of the nDNA wingless was performed using 3.0 µl genomic DNA of each individual in 12 µl reactions. Otherwise, the recipe was as for mtCOI. The PCR settings for COI amplification were as follows: an initial denaturation step at 95 °C for 1 min; 35 cycles of 20 s denaturation at 95 °C, 30 s annealing at 43 °C, product extension at 72 °C for 30 s; a final extension at 72 °C for 5 min. The PCR settings for the wingless amplification were identical except for the annealing temperature which was set to 50 °C.

**Table 1.** Information on the primers used to amplify COI mtDNA and Wingless nucDNA.

Primer	Direction	Sequence (5’ – 3’)	Gene	Reference
COI-F	forward	GGTCAACAAATCATAAAGATATTGG	COI	Folmer et al. 1994
COI-R	reverse	TAAACTTCAGGGTGACCAAAAAATCA	COI	Folmer et al. 1994
Wingnut1a	forward	GAAATGCGNCARGARTGYAA	wingless	Vitecek et al. 2015
Wingnut3	reverse	ACYTCRCARCACCARTGRAA	wingless	Vitecek et al. 2015

To verify the success of the amplification and to detect possible contamination, the PCR products and negative controls were visualized on a 1.5% agarose gel. Before sequencing, the PCR products were purified using an ExoSap purification. PCR products were then sequenced bidirectionally using the PCR primers on an ABI 3730XL sequencer at the Senckenberg Biodiversity and Climate Research Centre Laboratory Centre using standard reaction parameters.

### ﻿Sequence editing

Sequences of all gene fragments were edited and aligned in Geneious Prime v. 11.1.5. Primer regions were deleted from the final consensus sequences. Ambiguities in nuWG were coded following standard IUPAC codes. The sequences were aligned using default settings of the Geneious alignment tool and short sequences under 400bp were excluded from the alignment.

### ﻿Bayesian phylogenetic reconstruction

Two data sets were used for the Bayesian phylogenetic reconstruction: a 658 bp long alignment of 301 specimens sequenced for mtCOI, and a 472 bp long alignment of 196 specimen sequences for nuWG. The data sets were collapsed to unique haplotypes using the program Collapse 1.2 with default settings (Posada 2004). The best fitting evolution model of each data set was calculated using JModeltest v. 2.1.10 (Darriba et al. 2012). According to the AICc = AIC with correction for smaller sample sizes, BIC = Bayesian Information Criterion and DT = decision-theoretic performance-based criteria HKY+I+G was the best-fitting model for the COI data set, while K80+I+G was the best for the wingless data set.

The ultrametric tree was computed with BEAUti (Bayesian Evolutionary Analysis Utility) v. 1.10.4 and BEAST (Bayesian Evolutionary Analysis Sampling Trees) v. 1.10.4 (Drummond and Rambaut 2007). In BEAUti, the uncorrelated relaxed clock was chosen with a lognormal relaxed distribution for both data sets. The tree prior was set to “Speciation:Yule Process” with a random starting tree. For both data sets the priors kappa, pInv, and the substitution model, were adjusted as suggested in JModeltest. The COI dataset was run for 500,000,000 generations, sampling every 50,000^th^ tree and wingless for 100,000,000 generations, sampling every 10,000^th^ tree. To visualize and analyze the MCMC log files from BEAST, the MCMC Trace Analysis Tool (Tracer) v. 1.7.1 was used (Rambaut et al. 2018). Trees were combined in LogCombiner v. 1.7.1, the first 30% were thereby discarded as burn-in. With TreeAnnotator v. 1.10.4 a maximum clade credibility tree was generated and visualized in FigTree v. 1.4.

### ﻿Molecular species delimitation using GMYC

The General Mixed Yule Coalescent (GMYC) method was developed to delimit independently evolving species, using single locus data (Fujisawa and Barraclough 2013). This method defines species boundaries by identifying the transition point in the branching pattern of a haplotype ultrametric tree. This transition separates population-level processes, such as coalescence, from species-level processes, such as diversification (Pons et al. 2006). The GMYC analysis was conducted in R, v. 3.6.3 (R Core Team 2024), using the splits (Species Limits by Threshold Statistics) package (Ezard et al. 2017). The ultrametric trees were analyzed using the single-threshold method, which uses a single threshold to specify the transition from between- to within-species branching (Monaghan et al. 2009). This approach is considered more conservative than the multiple threshold method that allows different transition thresholds across the phylogeny. We hereafter refer to the GMYC derived species surrogates as molecular operational taxonomic units (MOTUs).

### ﻿Species abundances and environmental variables

We tested which environmental variables were associated with the abundance of the delimited MOTUs using Generalized Linear Models (GLMs). Prior to the analysis all environmental variables were tested for correlation using the Pearson correlation test. Every variable that had a significant correlation coefficient above 0.7 or below -0.7, was removed from the statistical analysis. Sampling sites for which measurements were not available were also removed. The GLM was implemented in the R package mvabund (Wang et al. 2012b) using the function manyglm. In this approach, a separate GLM is fit to each MOTU. The approach allows the use of non-normal data distributions, incorporating several explanatory variables simultaneously and takes correlation between species into account (Wang et al. 2012b). Because we collected many more specimens and often more species at some sites than others, we included the number of *Rhyacophila* individuals collected at each site as “individuals per site” to control for sampling biases. The analysis was run on raw MOTU count data, using the negative binomial distribution, which has been shown to be appropriate for count data because the mean-variance function tends to be quadratic rather than linear (O’Hara and Kotze 2010). To check if the assumptions (e.g., the distribution) of the model were correct, normal Q-Q, residuals vs fitted and Scale-Location plots were used. The final formula for the generalized linear model was: manyglm (species abundance matrix ~ Individuals per site + basin + elevation + conductivity + dissolved oxygen + Air temperature + ORP, family = negative binomial), all the continuous variables were scaled and centered, and basin was included as a factor. To test for significant effects of the explanatory variables on species abundance ANOVA was computed using the anova.manyglm function. We used a bootstrap method based on probability integral transform residuals, the “PIT-trap” (Warton et al. 2017), for estimating p-values were obtained based on 1000 resamples. In addition to these multivariate test statistics, univariate species-by-species results were calculated with p-values adjusted for multiple testing, using a step-down resampling procedure (Wang et al. 2012b). Level of significance was p < 0.05.

## ﻿Results

### ﻿Dataset

In this study, in total 524 *Rhyacophila* individuals (415 larvae and 109 adults) were included. For the *Rhyacophila* larvae samples, 301 COI sequences (658 bp, 75% success rate) and 196 wingless sequences (472 bp, 50% success rate) were generated. Furthermore, 109 sequences of adult *Rhyacophila*, which were collected from the same basins, were generated. The COI alignment contained 207 unique haplotypes, whereas the wingless alignment contained 150 unique haplotypes. The data is available in the Barcode of Life BOLD Systems database as record set Hengduan Shan Rhyacophila “HSRHY”.

### ﻿Species delimitation and life stage association

#### ﻿GMYC Analysis

The threshold time (T) infers the transitioning from speciation level events to coalescent level events. As expected T varied between the two gene fragments, transitioning earlier with the nuclear marker (T = -0.01), compared to the mitochondrial (T = -0.03) (Suppl. material 1). Transition points are indicated by red lines in the lineage-through-time plots and specimens belonging to the same GMYC species are marked red in the phylogenetic tree (Suppl. material 1). The tip nodes separating GMYC clusters and nodes within clusters were well supported (BBP > 0.95) in phylogenetic trees from both gene fragments. Deeper nodes, however, were found to have only weak support (BPP < 0.95) (Suppl. material 1).

The single threshold method delimited 66 putative species (hereafter referred to as MOTUs) composed of 42 distinct clusters and 24 singletons (represented by a single haplotype) in the COI dataset. GMYC analysis of the wingless gene fragment delimited 27 putative species, of which five MOTUs were singletons (Suppl. material 1). Several specimens associated with COI MOTUs clustered within different WG GMYCMOTUs, showing incongruence for species delimitation patterns among the gene fragments using GMYC. Since we successfully sequenced many more specimens for mtCOI, we performed all downstream analyses based on the COI-derived MOTUs.

### ﻿Larval abundance and distributional patterns

Overall, larval *Rhyacophila* were collected at 49 of 63 sampling sites, covering the four main river basins and an elevational range of 1022 m – 4381 m a.s.l. COI MOTUs were distributed as follows: 279 individuals (134 haplotypes, 47 MOTUs) in Yangtze, 67 individuals (39 haplotypes, 18 MOTUs) in Mekong, 39 individuals (20 haplotypes, 13 MOTUs) in Salween and 20 individuals (8 haplotypes, 7 MOTUs) in Irrawaddy.

**Figure 2.**
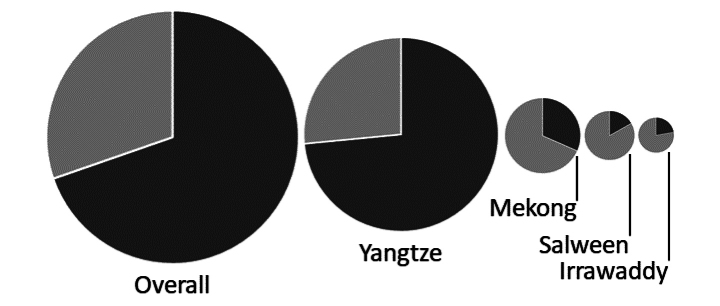
Distribution of shared (pale grey) and private (dark grey) mtCOIMOTUs at the river basin level. The size of the circle is relative to the number of mtCOIMOTUs found per basin.

On average we found four COI MOTUs per sampling site, with values ranging from one to twelve. The highest numbers of MOTUs occurred between 2000 m a.s.l. and 3500 m a.s.l., with site 61 at 2840 m a.s.l. in the Yangtze river basin exhibiting the greatest MOTU richness (*n* = 12). The Yangtze river basin was dominated by MOTU 20 (23 individuals, 12 different haplotypes). This MOTU contains adults that were identified as belonging to the *hingstoni* species group. The Mekong river basin was characterized by the MOTUs 13 (6 haplotypes) and 15 (7 haplotypes). In the Salween and Irrawaddy river systems, no single MOTU dominated the river basin. Many MOTUs (43 of 66) were rare and only occurred at one or two sampling sites. With the remaining MOTUs mostly occurring at three or four sites (max 8 sites), and the majority of MOTUs restricted to a single basin (46 of 66), few MOTUs can be considered widespread (Fig. 2).

**Figure 3.**
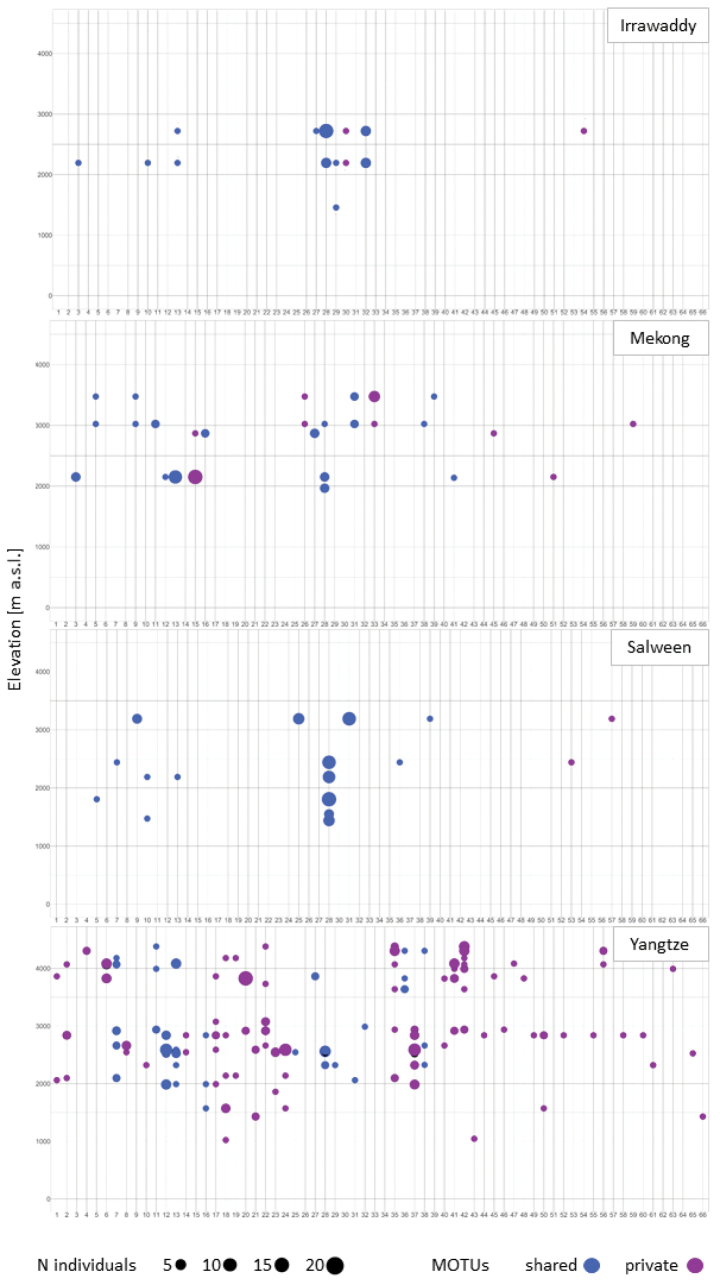
Elevational distribution of all recovered MOTUs. Shown are the elevation (y-axis) of the sites where each MOTU (x-axis) was found. The plot is segmented by basin to highlight the distribution of wide spread MOTUs vs those MOTUs that were private to an individual basin. Circle size reflects the number of individuals. Point color indicates distribution of MOTUs (shared among basin: blue; private: purple).

The distribution of COI-based MOTUs varied greatly between Yangtze and the three other river basins. In the Yangtze 74% of MOTUs were private, and were only collected in this catchment. The Mekong, Salween and Irrawaddy contained mostly abundant MOTUs that occurred in at least two basins (Figs 2, 3). Four MOTUs were especially abundant (sampled in three basins at up to eight sampling sites) and covered a wide range of environmental variables: MOTU 28 (Yangtze, Mekong, Salween), MOTU 31 (Yangtze, Mekong, Salween), MOTU 27 (Yangtze, Mekong, Irrawaddy) and MOTU 13 (Yangtze, Mekong, Salween).

Variability of environmental parameters is presented in Table 2. Because of the effect of elevation on other environmental variables, e.g. temperature, the wide altitudinal range may explain the variability of the other measured environmental variables in the Yangtze river basin.

**Table 2.** Environmental parameters presented as ranges for each river basin.

River Basin	Altitude (m a.s.l.)	Conductivity (µs cm^-1^)	Air Temp. (°C)	DO (mg/L)	ORP (mV)
Yangtze	1022–4381	7.3–286.5	6.7–23.7	6.88–8.85	62.5–303.2
Mekong	1966–3475	28.3–205.1	10.2–19.8	7.65–8.02	98.2–152.1
Salween	1439–3190	9.5–60.4	15.6–21.1	7.36–7.69	98.6–152.3
Irrawaddy	1457–2721	15.6–32.7	14–21.3	7.51–7.99	111.8–162.3

Multivariate regression analysis using Generalized Linear Models (GLMs) were applied to test whether environmental variables were significantly associated with the occurrence and abundance of delimited MOTUs. Prior to GLM analysis, Pearson correlation tests revealed strong and significant correlations of elevation with water temperature as well as of conductivity with salinity, total dissolved solids and the pH. Hence, water temperature, total dissolved solids, salinity, and pH were not included in the GLM analysis. Explanatory variables used in the GLM analysis all exhibited significant effects on mean MOTU abundance (Table 3). Besides individuals per site (Deviance = 144.8, *p* = 0.001), which reveals potential sampling biases, river basin (Deviance = 176.4, *p* = 0.003) and oxidation reduction potential (Deviance = 108, *p* = 0.001) were the most important variables.

**Table 3.** Analysis of deviance table for the multivariate Generalized Linear Model for the COI gene using the MOTUs delimited with the GMYC approach.

	Residual degrees of freedom	Deviance	*p*
Individuals per site	44	144.8	0.001***
River Basin	41	176.4	0.003**
Elevation	40	78.5	0.001***
Conductivity	39	88.2	0.004**
DO	38	82.3	0.005**
Air Temperature	37	71.2	0.009**
ORP	36	108.0	0.001***

In addition to multivariate tests, which examine the influence of each environmental variable used in the GLM on mean MOTU abundance, univariate tests were conducted. These examine the effect of each variable on each MOTU individually. Univariate species analysis revealed a large range of responses to the different environmental variables used in the GLM analysis. Statistically significant responses were found in the COI dataset with the following GMYCMOTUs: MOTU 31 exhibited a significant response towards conductivity (Deviance = 16.57, *p* = 0.037). This MOTU occurred at middle altitudinal sites (2322 m – 3190 m), with conductivities ranging from 20 µs per cm to 79.7 µs per cm indicating the importance of a relatively low conductivity for the abundance of this MOTU (Suppl. material 2). For MOTU 20, a statistically significant response towards the oxidation reduction potential was found (Deviance = 15.81, *p* = 0.05). This MOTU dominated the Yangtze with 23 individuals in 12 haplotypes at two sampling sites (2918 m with an ORP of 78.9 mV and 3827 m with an ORP of 162.1 mV; Fig. 3).

**Figure 4.**
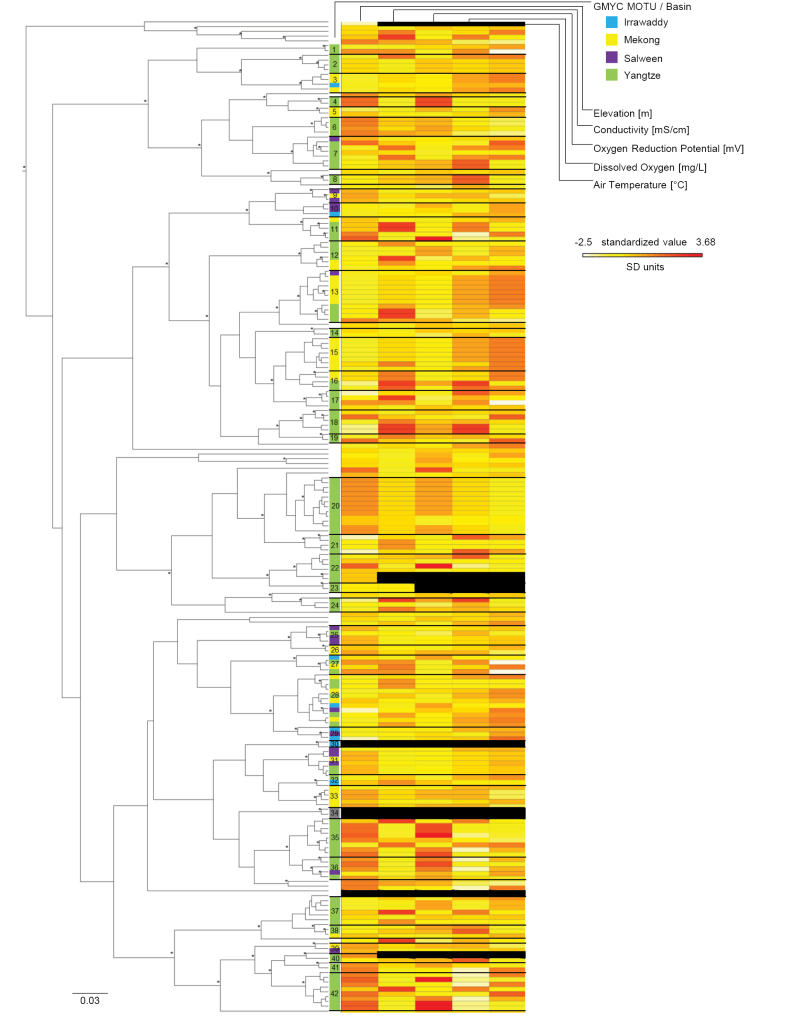
Bayesian maximum clade credibility tree for the COI gene. Vertical bars on the right of the tree indicate GMYC delimited MOTUs. Colors of the bars indicate the basin where the individual was sampled. Asterisks on the tree mark nodes with a posterior probability > 0.95. The heatmap on the right presents standardized values for elevation, conductivity, dissolved oxygen in mg per liter in the water, oxidation reduction potential, and air temperature. Black bars in the heatmap indicate missing values.

MOTU composition varied along the environmental gradients within and between basins (Figs 3, 4, Suppl. material 2). Some haplotypes of the same MOTU seem to share environmental preferences, indicating distinct environmental niches. Especially the oxidation reduction potential (Fig. 4, center bars in the heat map) and elevation (Figs 3, 4, left bars of the heat map) showed patterns along the phylogenetic tree.

## ﻿Discussion

*Rhyacophila* from the Hengduan Mountains were sequenced to investigate the species diversity of this region. We linked the delimited MOTU diversity to habitat characteristics and environmental gradients. This study revealed primary structuring of species occurrences aligns with river basins, with numerous unique MOTUs identified in the Mekong and Yangtze basins. Additionally, MOTU variation was associated with ORP followed by conductivity. Conductivity is indicative of geological and geomorphological conditions and also appears to have a structuring effect. Furthermore, elevation and the correlated parameters, air temperature, and DO also showed a significant structuring effect. We will first discuss methodological limitations of the study before discussing which topographic and/or environmental drivers may be structuring *Rhyacophila* species in the Hengduan Mountains biodiversity hot spot.

### ﻿Sampling

Unbalanced geographical sampling can influence the success of species associations by inducing oversplitting, because geographically more distant haplotypes may increase intraspecific divergence (Talavera et al. 2013). The fact that the majority of sampling sites are located in the Yangtze river basin (33 (67%)) compared with seven in Salween, six in Mekong, and three in Irrawaddy could have influenced species associations and led to overestimating the basin as the primary structuring variable. Additionally, the Yangtze sampling also covers the most diverse environmental conditions (greater elevational range, thermal variation, and conductivities). Again, the greater environmental variation could have fostered greater diversity being associated with the basin level.

Most of the delimited MOTUs here were rare, containing only one to five individuals (73%). This may result in a bias for the multivariate analysis where “Individuals per Site” was found to be strongly significant. However, “rare” species are a common feature in ecological communities (Cao et al. 1998; Flather and Sieg 2007; Brasil et al. 2017; Wells et al. 2019). Biological communities often comprise a few very abundant and extensively distributed species, which dominate the biomass and stand in contrast to the majority of species that are rare, exhibit a restricted geographic range, and contribute to overall biodiversity (Kunin and Gaston 1993; McGill et al. 2007; Flather and Sieg 2007; Gaston 2008; Magurran and Henderson 2010). Our results are in line with this pattern. How rare species should be dealt with in community ecology studies is still under discussion. Some studies suggest deleting rare species from the dataset altogether to reduce “background noise” in statistical analysis (Austin and Greigh-Smith 1968; Boulton et al. 1992). Others state there is no good biological reason to delete rare species and doing so will lead to a loss of valuable ecological information that is crucial for correct analysis and interpretation of the community (Goodall 1969; Slocomb and Dickson 1978; Cao et al. 1998). Extensive temporal sampling could better elucidate the proportions of rare and common *Rhyacophila* species in the study region, but this is far beyond the scope of this study. Based on the single sampling period we analyzed, we did not exclude rare species as the study aimed to uncover the mainly unknown diversity of the Hengduan Mountains and was not a study on community ecology. We thus expected many rare and endemic lineages as previously reported in general (Gerhold et al. 2015), and in caddisflies in particular (Múrria et al. 2012, 2018; Hoppeler et al. 2016).

### ﻿Genetic markers

The mitochondrial marker COI has been widely used in DNA barcoding and species delimitation studies (Hebert et al. 2003; Pons et al. 2006; Zhou et al. 2007). A key finding of this study is that the nuclear wingless signal was sufficiently variable to differentiate taxa and even exhibited minimal intraspecific variation. However, the signal was not congruent with the mitochondrial COI in the phylogenetic reconstruction. This is in contrast to the result from nuCAD, which was congruent with COI data but resulted in a much lower resolution of species relationships in *Himalopsyche* (Hjalmarsson et al. 2018). Wingless was recently used to resolve the shallow phylogenetics of a *Wormaldia* species complex in central Europe (Mewis et al. 2024). Together with our study it is likely that both nuclear genes WG and CAD are good candidates to combine with COI to assess shallow interspecific or intraspecific variation in caddisflies.

### ﻿*Rhyacophila* co-occurrence

On average, five MOTUs were found co-occurring per site, but total numbers ranged from one to twelve, with the highest levels of α-diversity occurring between 2000 m a.s.l. and 3500 m a.s.l. Larvae of most *Rhyacophila* species are predators, hunting other freshwater invertebrates, whereas a few species are herbivores or shift from herbivorous to carnivorous in different instar stages (Smith 1968; Thut 1969; Lavandier and Céréghino 1995; Céréghino 2002; Elliott 2005). Larvae of the genus *Rhyacophila* have been reported to coexist because partitioning of their habitats allows them to use their habitat more efficiently (Lavandier and Céréghino 1995; Taira and Tanida 2011). An extreme case was recently reported from Japan with nine and ten *Rhyacophila* species co-occurring in two mountain streams (Taira 2023). In those sites, differences in vertical microhabitat use in the hyporheic zone, microhabitat preferences as well as life history variation likely facilitate co-existence of species. In our study area, the life history and ecology of *Rhyacophila* species are unexplored, but with up to twelve potential species co-occurring at single sites, this warrants in-depth studies on the drivers of co-existence.

### ﻿Larval abundance and environmental gradients

MOTU abundance exhibited significant responses to all examined river basins and environmental variables (elevation, conductivity, dissolved oxygen, air temperature and oxidation reduction potential). According to our GLM the most important physical variable was river basin (Deviance = 176.4, *p* = 0.003) followed by the physico-chemical variables oxidation reduction potential, conductivity, and elevation. Elevation integrates a number of physico-chemical parameters as well as hydromorphological features that are likely to influence the microscale occurrence of benthic invertebrates in general. Catchment structure and the surrounding topography, on the other hand, dictate the primary limits of out-of-stream dispersal of aquatic insects. While we cannot conclusively infer the order of importance, it seems reasonable that catchment is the most important feature structuring the community of *Rhyacophila* species in the Hengduan Mountains, followed by instream parameters.

Generally, elevation is known to be a major organizational gradient of biodiversity, especially in high mountainous areas (Rahbek 1995; Lomolino 2001; Baniya et al. 2010; Wang et al. 2012a). The influence of altitude on distributional patterns of freshwater invertebrates (Min and Kong 2020; Myers et al. 2021) and Trichoptera (García and Ferreras-Romero 2008; Hoppeler et al. 2016; Tonkin et al. 2017; Ríos-Touma et al. 2022) has been reported in various ecosystems all over the world. Larvae of the genus *Rhyacophila* generally prefer fast-flowing river conditions and are very abundant in headwaters and high altitudes (Smith 1968; Thut 1969; Pauls et al. 2023; Taira 2023). It is thus somewhat surprising that in the results presented here, elevation was only as a secondary explanatory variable, although *Rhyacophila* larvae occurred at both low and very high altitudes (1022–4381 m a.s.l.). It is, however, important to mention that elevation integrates a gradient of various other chemical and physical environmental variables (e.g., water temperature, conductivity, dissolved oxygen, flow velocity, channel type) that change with altitude and more directly influence the dispersal of freshwater invertebrates (Jowett and Richardson 1990; Jacobsen 2008; Min and Kong 2020). The strong correlation of elevation with water temperature and the relative amount of dissolved oxygen in the water supports this fact.

In our study, some MOTUs exhibited restricted environmental preferences but were distributed over a large geographical scale. Keeping the study’s somewhat limited sampling in mind, these patterns may reflect specialization of individual MOTUs to specific environmental conditions. For example, MOTUs 12 and 13 were sampled in various basins but only at middle altitude between ~2000–2600 m a.s.l., while MOTU 31 seems associated with very low conductivity values in different river basins. In contrast, MOTU 35 was sampled only in the Yangtze, but from sites between 2098 m and 4381 m a.s.l. across a range of physico-chemical conditions. More extensive sampling and analyses are required to fully understand if and which of the region’s *Rhyacophila* species exhibit signatures of environmental specialization.

The highly differentiated community compositions between Yangtze and the other three river systems is most likely due to landscape barriers that inhibit migration and drive allopatric speciation (Bilton et al. 2001; Múrria et al. 2013). Hoppeler et al. (2016) found high turnover at a very small geographical scale within four Nepalese river systems, with 63% of the potential Hydropsychidae species being confined to one river system. The authors argued that the high mountains and deeply incised valleys of the Himalaya constitute a significant barrier for migration and therefore restrict dispersal to within catchment movement, potentially driving higher numbers of potentially catchment-endemic species. Our findings of high heterogeneity in species composition among especially high altitudinal habitats also suggest that these isolated areas harbor many microendemic species, many of which likely remain unknown. More work is needed to examine and preserve this diversity in the light of the ongoing environmental change (Parmesan 2006; Shrestha and Devkota 2010; Bálint et al. 2011; Shah et al. 2012, 2015; Finn et al. 2013).

## ﻿Outlook and conclusions

Approximately 41% of insects are already in decline, with especially high numbers occurring among aquatic insects, making their decline twice as fast as that of vertebrates (Sánchez-Bayo and Wyckhuys 2019). Mountain communities are particularly endangered, because changes in temperature and precipitation strongly affect their hydrology, morphology, and physico-chemical conditions at small geographic scales (Jacobsen et al. 2012; Immerzeel et al. 2013; Shah et al. 2015). For example, the Himalaya has been reported to warm three times faster than the world average (Xu et al. 2009) and is predicted to experience an increase in rainfall frequency and intensity (Shrestha and Devkota 2010). Aquatic insects are expected to migrate to higher altitude habitats on account of the rising temperatures, leaving endemic headwater species, also present in this thesis, without a climatically suitable habitat as they get stuck in a “summit trap” (Bálint et al. 2011; Shah et al. 2012).

In particular, the biodiversity hotspot of the Hengduan Mountains in China lacks research about the ecology and biodiversity of the regional insect fauna (Myers et al. 2000; Dudgeon et al. 2006; Liu et al. 2020). To address this knowledge gap, an increased effort to study the region’s aquatic insects is necessary. The DNA taxonomy method used here to assess biodiversity without extensive morphological taxonomic work may be a potential first step. However, this needs to be followed-up by morphological and ecological studies. An increased effort on ecological, taxonomic and faunistic work using caddisfly larvae and adults is necessary to improve our understanding of caddisfly diversity in the region, enable life-stage associations and promote a more conclusive integrative taxonomy of *Rhyacophila* and other caddisflies in the Hengduan Mountains.

## ﻿Additional information

### Use of AI

No use of AI was reported.

### Author ORCIDs

Steffen U. Pauls https://orcid.org/0000-0002-6451-3425

### Data availability

The specimen, collections and sequence data from this study are publicly available in the BOLD Systems Data Warehouse under https://portal.boldsystems.org/result?query=HSRHY[recordsetcode].

## Supplementary materials

Supplementary material 1 Results of GMYC Analyses Data type pdf https://binary.pensoft.net/file/1488183This dataset is made available under the Open Database License (http://opendatacommons.org/licenses/odbl/1.0/). The Open Database License (ODbL) is a license agreement intended to allow users to freely share, modify, and use this Dataset while maintaining this same freedom for others, provided that the original source and author(s) are credited.Sonja Gerwin, Xiling Deng, Fengzhi He, Steffen U. Pauls

Supplementary material 2 Distribution of MOTUs in relation to conductivity Data type pdf https://binary.pensoft.net/file/1488184This dataset is made available under the Open Database License (http://opendatacommons.org/licenses/odbl/1.0/). The Open Database License (ODbL) is a license agreement intended to allow users to freely share, modify, and use this Dataset while maintaining this same freedom for others, provided that the original source and author(s) are credited.Sonja Gerwin, Xiling Deng, Fengzhi He, Steffen U. Pauls
