# Supplementary material for: ﻿Diversity of Rhyacophila (Trichoptera, Rhyacophilidae) in the Hengduan Mountains
Source: Zookeys. 2025 Dec 10;1263:69–88. doi: 10.3897/zookeys.1263.153111 (PMC12712619; doi:10.3897/zookeys.1263.153111)
Supplement: Supplementary material 1 — Results of GMYC Analyses [file zookeys-1263-069_article-153111__-s001.pdf]

Supplementary Material A

Results of GMYC Analyses

Table SMA: GMYC analysis output for COI and *wingless*. Presented are: T= threshold genetic distance from the branch tips where transition occurred, N_{GMYC}= the number of delimited groups and their confidence intervals (CI), L_0=likelihood of null Modell, L_{GMYC}=likelihood of GMYC models and the significance of the likelihood ratio (LR).

	T	N_{GMYC}	(CI)	L_0	L_{GMYC}	LR
COI	-0.03	66	52-86	1399.98	1406.68	0.001**
Wingless	-0.01	27	4-37	1138.14	1140.59	0.09 n.s

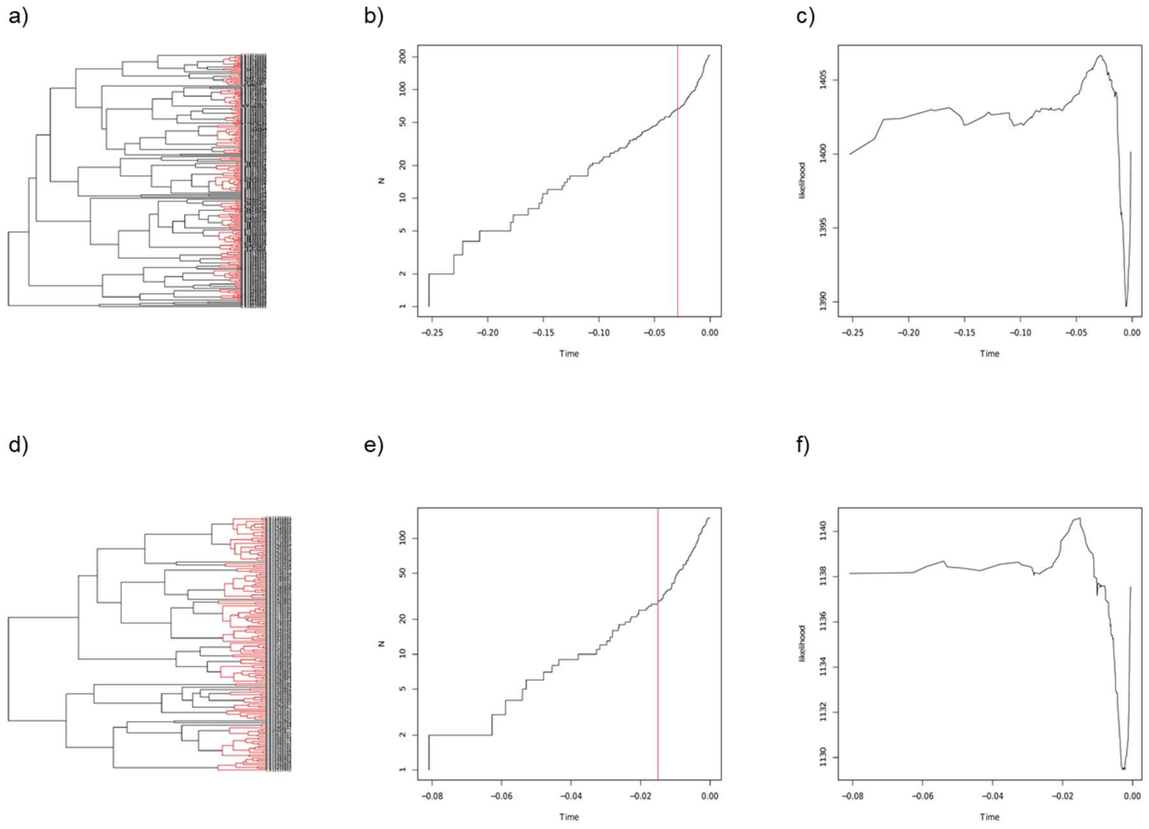

Figure SMA: Output of the GMYC analysis for a) - c) COI and d) - f) *wingless*. Specimens marked red in the phylogenetic trees (a) and d)), belong to the same GMYC-species. The red lines in the lineage-through-time plots in the centre (b) and e)), mark the points of transition between the branching patterns. The peaks in the likelihood-through-time plots on the right (c) and f)), indicate the maximum likelihood point, where the border between inter- and intraspecific branching patterns is expected.

The tip nodes separating clusters and the ones within a cluster were generally well supported (BBP>0.95) in phylogenetic trees from both gene fragments. More basal nodes, however, were found to have only weak support and low Posterior probability values (BPP<0.95) (Figure 10).
