# Supplementary material for: ﻿Diversity of Rhyacophila (Trichoptera, Rhyacophilidae) in the Hengduan Mountains
Source: Zookeys. 2025 Dec 10;1263:69–88. doi: 10.3897/zookeys.1263.153111 (PMC12712619; doi:10.3897/zookeys.1263.153111)
Supplement: Supplementary material 2 — Distribution of MOTUs in relation to conductivity [file zookeys-1263-069_article-153111__-s002.pdf]

Supplementary Material B

Distribution of MOTUs in relation to conductivity

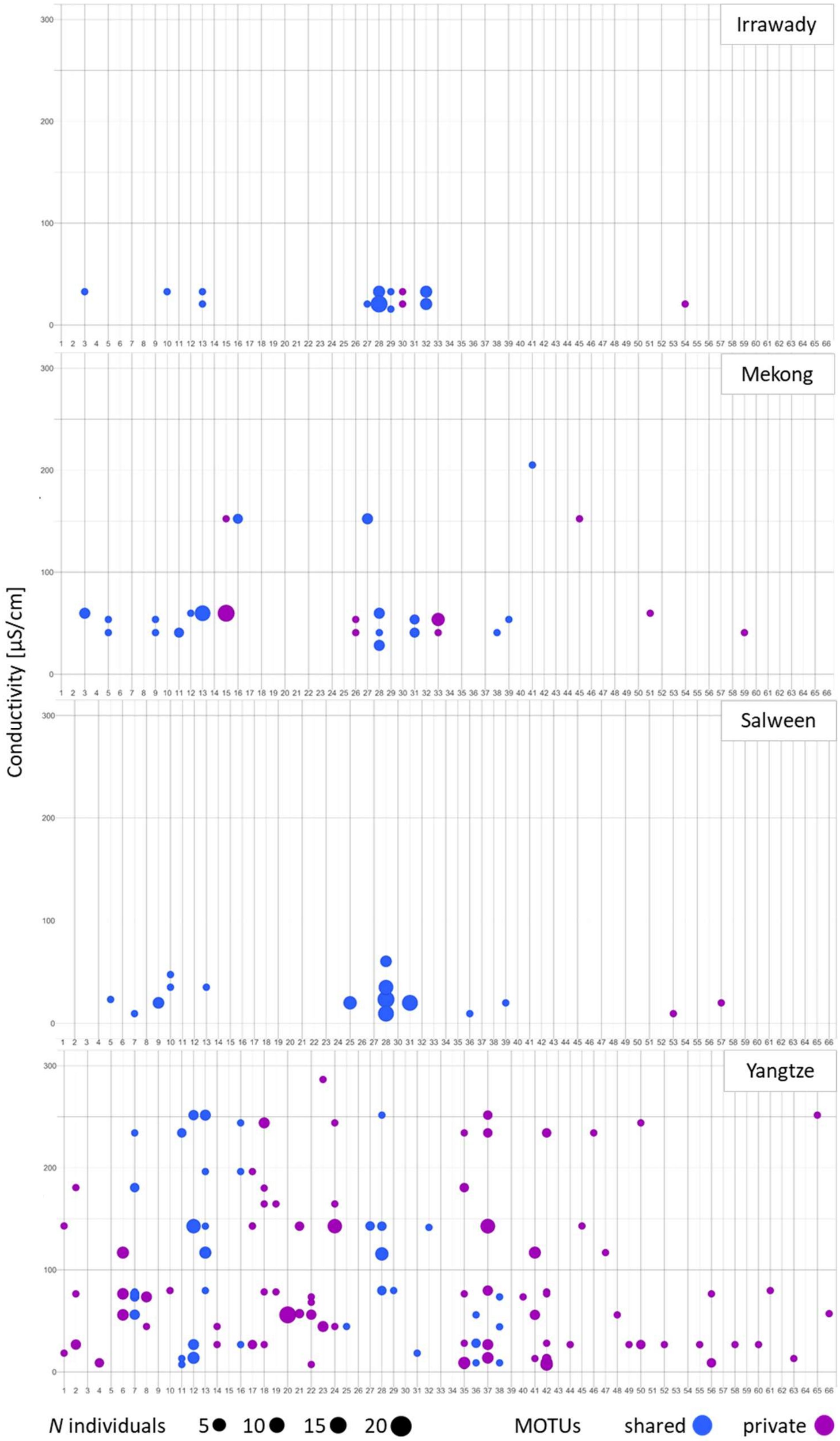

Scatterplots of MOTUs from the sampled river basins against conductivity. Circle size reflects the number of individuals. Point color indicates distribution of MOTUs (shared among basin: blue; private: purple).
